# Changes in Metabolism and Content of Chlorophyll in Common Duckweed (*Lemna minor* L.) Caused by Environmental Contamination with Fluorides

**DOI:** 10.3390/molecules29102336

**Published:** 2024-05-16

**Authors:** Jan Kamiński, Alicja Stachelska-Wierzchowska, Dariusz J. Michalczyk, Agnieszka Klimkowicz-Pawlas, Ewa Olkowska, Lidia Wolska, Agnieszka I. Piotrowicz-Cieślak

**Affiliations:** 1 Department of Plant Physiology, Genetics and Biotechnology, University of Warmia and Mazury, Oczapowskiego Str. 1A, 10-719 Olsztyn, Polanddarim@uwm.edu.pl (D.J.M.); 2 Department of Physics and Biophysics, University of Warmia and Mazury, Oczapowskiego Str. 4, 10-719 Olsztyn, Poland; alicja.stachelska@uwm.edu.pl; 3 Department of Soil Science Erosion and Land Protection, Institute of Soil Science and Plant Cultivation—State Research Institute, Czartoryskich Str. 8, 24-100 Puławy, Poland; agnes@iung.pulawy.pl; 4 Department of Environmental Toxicology, Faculty of Health Sciences, Medical University of Gdansk, Dębowa Str. 23A, 80-204 Gdansk, Poland; ewa.olkowska@gumed.edu.pl (E.O.); lidiawolska@gumed.edu.pl (L.W.)

**Keywords:** chlorophyllase, aminolevulinic acid dehydratase, fluoride, chlorophyll absorption spectra, intensive poultry farming

## Abstract

The impact of fluorine on plants remains poorly understood. We examined duckweed growth in extracts of soil contaminated with fluorine leached from chicken manure. Additionally, fluorine levels were analyzed in fresh manure, outdoor-stored manure, and soil samples at varying distances from the manure pile. Fresh manure contained 37–48 mg F^−^ × kg^−1^, while soil extracts contained 2.1 to 4.9 mg F^−^ × kg^−1^. We evaluated the physiological effects of fluorine on duckweed cultured on soil extracts or in 50% Murashige–Skoog (MS) medium supplemented with fluorine concentrations matching those in soil samples (2.1 to 4.9 mg F^−^ × L^−1^), as well as at 0, 4, and 210 mg × L^−1^. Duckweed exposed to fluorine displayed similar toxicity symptoms whether in soil extracts or supplemented medium. Fluoride at concentrations of 2.1 to 4.9 mg F^−^ × L^−1^ reduced the intact chlorophyll content, binding the porphyrin ring at position 3^2^ without affecting Mg^2+^. This reaction resulted in *chlorophyll a* absorption peak shifted towards shorter wavelengths and formation of a new band of the F^−^-*chlorophyll a* complex at λ = 421 nm. Moreover, plants exposed to low concentrations of fluorine exhibited increased activities of aminolevulinic acid dehydratase and chlorophyllase, whereas the activities of both enzymes sharply declined when the fluoride concentration exceeded 4.9 mg × L^−1^. Consequently, fluorine damages *chlorophyll a*, disrupts the activity of chlorophyll-metabolizing enzymes, and diminishes the plant growth rate, even when the effects of these disruptions are too subtle to be discerned by the naked human eye.

## 1. Introduction

Many human activities result in environmental pollution with pesticides, herbicides, artificial fertilizers, antibiotics, and various other substances, which have toxic effects on animals and plants [1,2]. Equally harmful are some naturally occurring pollutants and human activities can increase their concentrations excessively. These environmental toxins include fluorine and its compounds [3].

Fluorine is the 13th most abundant element in the Earth’s crust. Its average concentration in continental plates is as high 557 mg × kg^−1^ [4]. Fluorine anions and their compounds, fluorides, are commonly found in groundwater and soil, especially in clay soils. The bedrock can sometimes be a natural source of soil fluoride. Fluoride-containing rocks include granite, syenites, gneisses, and hornblende [5,6,7,8,9]. The concentration of F^−^ in soil at the site of gneisses and granites exceeds 5000 mg × kg^−1^ [2]. Through weathering of fluoride-containing rocks, F^−^ enters soils and groundwater. Volcanic eruptions could be another natural source of F^−^ [2]; however, they do not occur at this site. Fluorine escapes from volcanoes as HF gas, which is partly adsorbed onto volcanic ash. Through the volcanic route, an estimated 0.5 ± 0.2 Mt of fluorine enters the atmosphere annually [10]. However, none of these factors were a source of fluoride in the analyzed soil.

Fluorine present in the environment has anthropogenic origins in addition to the natural ones. The production of aluminum is one of the most important anthropogenic sources [11,12,13]. The production of 1 tonne of aluminum results in the release of 0.5–0.6 kg F [14]. Coal combustion greatly contributes to the release of F to the atmosphere [15]. Hard coal contains fluorine in amounts ranging from 4 to 40 g × kg^−1^ [1]. Annual HF emissions from coal-fired power plants in 2001 were as high as 55.8 million tonnes [16]. The increased level of fluorine in the environment is also due to the production of bricks and ceramic tiles [15]. This sector of industrial production releases 1.8 million tonnes F annually [17]. 

Fluorine compounds have been detected in groundwater in widely varying amounts depending on the country and sampling location. Worldwide, its content can range from 0.01 to 48 mg × L^−1^ [18]. In many African countries, China, South Asia, and the Middle East, groundwater is one of the main sources of drinking water [2,19]. According to WHO, the highest acceptable level of F in drinking water is 1.5 mg × L^−1^. Values above this level have been detected in many countries in North and South America and Eurasia. In Pakistan, the fluorine content is 7.85 mg × L^−1^ [20], 2.3 mg × L^−1^ in Algeria [21], about 5 mg × L^−1^ in Argentina [22], 1.38 mg × L^−1^ in Poland at an industrial waste site [23], 0.3–9.2 mg × L^−1^ in Greece at aluminum processing plants [24], 3.2 and 6.5 mg × L^−1^ in the USA at industrial sites in Pennsylvania, and 7 mg × L^−1^ in Estonia in aquifers of Silurian and Ordovician age [25].

Fluorine compounds are commonly used in agriculture. Fluorine-containing compounds constitute 53% of all agrochemicals and insecticides represent over 70% of them [26]. Some fluorinated insecticidal agents are widely used against flies or other insects in poultry houses. These include bifenthrin, indoxacarb [27,28], cyfluthrin, fipronil, and lambda-cyhalothrin [26,29,30]. 

Intensification of agricultural production plays a significant role in the increase in F^−^ content in soil and groundwater. The use of phosphate fertilizers [15], pesticides, fumigants, and fluoride-containing antibiotics increases the fluorine level in the environment [1,31,32]. Rainfall contaminated by emissions from phosphate fertilizer production contains more than 1 mg × L^−1^ of fluoride and this concentration is observed at a distance of up to 2 km from the emission source [33]. Fluoroquinolones are fluorine-containing antibiotics commonly used in poultry husbandry. The presence of fluoroquinolones has even been found in broiler meat. Of the fluoroquinolones analyzed (ciprofloxacin, norfloxacin, enrofloxacin, sarafloxacin, ofloxacin), ciprofloxacin was present in the highest amounts, exceeding 1 mg × kg^−1^ of poultry meat [34]. However, the content of other antibiotics can also be significant. The levels of enrofloxacin and norfloxacin in broiler meat exceeded 242 and 113 μg × kg^−1^, respectively [32,35,36,37,38]. As can be seen above, agricultural production leads to fluorine contamination of soil, water, and fertilizers through various pathways.

Fluorine present in the environment has extensively documented phytotoxic properties. Fluoride affects plants on many levels, including external morphology, tissue structures, and intracellular structures. Fluorine decreases the rate of cell division and growth, which hampers the overall plant growth and development [39]. Cell growth was markedly inhibited, for instance, in *Arabidopsis thaliana* cell suspension treated with 1 mM NaF [40]. Internal structure of cells gradually deteriorates with exposure time. This involves expansion and aggregation of endoplasmic reticulum, mitochondria losing electron density of the matrix, and eventual breakdown of tonoplast. Cells lose their shape and eventually collapse [41,42,43,44,45]. Fluoride toxicity in plants is also manifested by damage of cell membranes [46]. Fluoride stress induces lipoxygenase activity, which oxidizes membrane lipids and eventually causes electrolyte leakage. The significant role of membrane lipid oxidation in F stress is also indicated by increased malondialdehyde content [47,48] and increased amounts of small molecular antioxidants (ascorbate, GSH) were also detected in plants responding to the fluoride-induced stress [47,49,50,51].

Fluoride ions interact with proteins, binding to their amino acid side chains, interfering with protein folding, modifying the protein 3D structures, and disrupting their functions. Moreover, increase in ROS resulting from F-induced stress causes carbonylation of amino acids and permanently damages proteins. F^−^ ions form complexes with metal cations essential in enzyme function, like Mg, Mn, and Fe, which inhibits many enzymes [45]. Fluoride shows high affinity to Al, and F is often absorbed by plants in complexes with Al. It was shown that the AlF_4_^−^ complex acts as an analogue of the phosphate anion, thus inhibiting enzymes involving PO_4_^3−^, like ATPases and G proteins [52]. It is important, as enzymes involving phosphate are essential in all aspects of cell biology, including energy metabolism, signaling, and biosynthesis of various substances. Metal complexation by F interferes with other aspects of physiology, like Ca-related signaling.

Loss of chlorophyll is another widely documented and often very noticeable symptom of fluoride stress. Chlorosis usually begins at the leaf margins, progressing between veins towards the leaf base [1,3]. Leaves exposed to gaseous HF also show tissue damage, progressing over exposure time. Fluoride-induced leaf damage first appears in the spongy mesophyll and lower epidermis, followed by chloroplast damage in the palisade parenchyma [45,53]. Chlorosis is among the most universal symptoms reported in plants exposed to F^−^ contamination [54,55]. The mechanism of chlorophyll loss due to F stress is poorly understood. Complexation of Fe by F impairs chlorophyll biosynthesis [54]. Gaseous HF produces an acidic environment, which dissociates magnesium from the chlorophyll porphyrin ring. Both *chlorophyll a* and *chlorophyll b* levels decrease as a result of stress. However, both in plants growing under optimum conditions and plants subjected to stress, *chlorophyll a* usually clearly predominates in quantity [56,57].

F stress and disturbances in structure and contents of chlorophyll resulting from it not only impair light absorption by the photosynthetic apparatus but they also affect the photosynthetic electron transport chain. Total quantum efficiency of PSII is reduced due to inhibited Hill reaction. Fluoride ions substitute Cl^−^ in PSII, which blocks water photolysis and generates ROS [58]. Fan et al. [59] explored electron transport chain efficiency in tall fescue under combined F and Al stress. The PSI saturation with electrons dropped, while transferring electrons from plastoquinone increased. Photosynthesis impairment is also related to lowered activity of carbon-fixing and sugar-processing enzymes, like rubisco, amylase, invertase, and sucrose synthase [41,46,54]. Virtually nothing is known about reactions of fluoride ions with the carbon skeleton of chlorophyll. 

The aim of this study was to evaluate the impact of extracts of soil contaminated with manure from intensive poultry farming (and consequently contaminated with fluoride) on plant physiological state. Duckweed (*Lemna minor* L.) was used as a bioindicator plant and the following parameters were analyzed: fluoride and *chlorophyll a* content, rate of plant growth, activities of chlorophyllase, and aminolevulinic acid dehydratase. In addition, we studied the molecular mechanism of fluoride’s effect on chlorophyll in plants. To the best of our knowledge, the detailed mechanism of fluoride-induced chlorophyll decay in plants has not been fully understood to date.

## 2. Results and Discussion

### 2.1. Effect of Soil Extracts on the Growth of Duckweed (Environmental Samples)

**Figure 1:**
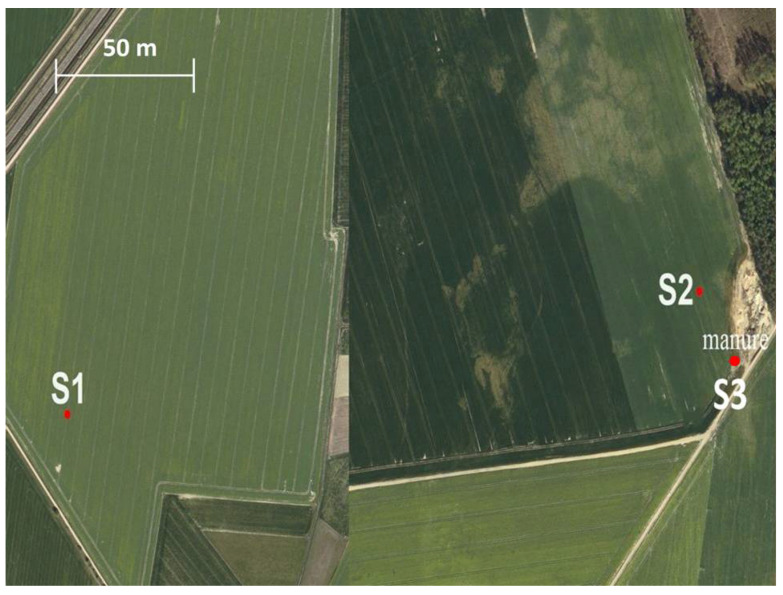
Place of sampling and location of manure pile.

Fluoride content was determined in three soil samples from fields located different distances from the improperly managed poultry manure pile. The location of the sampling points is shown in Figure 1. Soil sampling point 3 was located closest to the place where the manure from the poultry house was stored. It was shown that the highest fluoride content was in soils 2 and 3 and was 4.19 and 4.9 mg × kg^−1^ soil, respectively. A twice lower content of 2.1 mg × kg^−1^ was found in soil 1, located at a considerable distance from the manure pile (Table 1). The assessed soil composition (soils 1, 2, 3) did not differ significantly. Soils 1 and 2 were classified as sandy loam, while soil 3 was a loamy sand. More detailed data on soil properties are given in Table S1. 

The fluoride content in manure collected from the inside of the poultry house was 37–48 mg × kg^−1^ and the F**^−^** content in manure collected from the pile outside the chicken house was 1 mg × kg^−1^.

The analyzed soils had a relatively low Ca^2+^ content, and total aluminum was at the level of 4.2, 5.9, and 4.7 g × kg^−1^ (in soils 1, 2, and 3, respectively). Ca^2+^ and Al^3+^ contents were analyzed because these cations have a high affinity for F**^−^**, resulting in high F retention in clay soils rich in Al and Ca [15,17]. The fluorine content of the soil is also significantly affected by the soil pH. Under alkaline pH conditions, F mobility in the soil increases [60,61]; the same pattern (higher F solubility) is also observed at pH below 5 [62]. It should be emphasized that the soils analyzed were acidic (Table 1). In addition, fluoride-containing groundwater usually has a high pH and a high content of HCO_3_^−^ and Na^+^ [5,63,64,65]. The soil parameters analyzed (Table 1 and Supplementary Table S1) clearly indicate that the fluoride in analyzed samples was not a natural component of those soils but was leaked from the manure. Moreover, the manure stored inside the chicken house contained nearly fifty times more fluoride than the manure stored outside the chicken house. Apparently, much of the initial content of fluorides in manure is gradually leaked out due to atmospheric factors. 

Selecting the location of sampling points, we ruled out areas located close to industrial centers, as Singh et al. [1] indicated that proximity to industrial plants can also be a source of soil fluoride. Only the manure pile could be the source of fluorides in the soil studied in this paper. It should be clearly stated that the source of fluoride ions in poultry excrement is unknown. One can speculate that they could originate either in preparations used to combat pests in broiler houses or medicinal substances containing fluorine. 

Common duckweed was grown in soil extracts (samples taken from field at sites 1, 2, 3). We chose the duckweed as it is widely used as an indicator plant for the assessments of effects of chemical pollutants on plant physiology and biochemistry. Previous studies have mainly focused on heavy metal ions, especially Pb and Cd [66,67]. Common duckweed has not been investigated for responses to fluoride contamination to date. 

**Figure 2:**
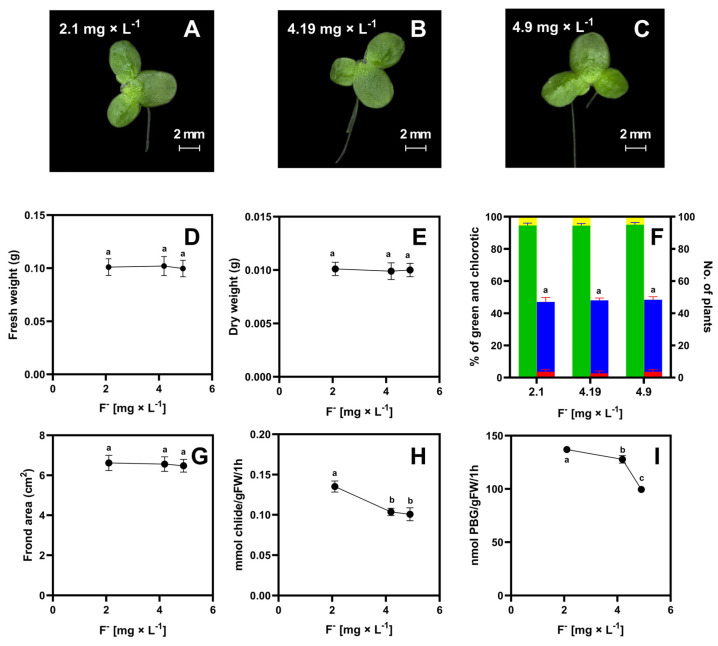
General appearance of plants after 7 days of growth in soil extracts (initial number of plants was ten)—(**A**–**C**); fresh weight [g]—(**D**); dry weight [g]—(**E**); percent frequency of green plants (green bar), percent frequency of yellow fronds (yellow bar), number of green plants (blue bar), number of yellow plants (red bar)—(**F**); surface area [cm^2^]—(**G**); chlorophyllase activity [mmol of chlorophyllide produced per 1 g of fresh weight within 1 h]—(**H**) and aminolevulinic acid dehydratase activity [as nmol of porphobilinogen produced per 1 g of fresh weight within 1 h]—(**I**). Tests were conducted in triplicates. The one-way analysis of variance (ANOVA) was carried out using Tukey’s post hoc test (*p* ≤ 0.05). Small letters represent groups of significant difference.

Across all of the soil extracts analyzed, it was shown that the duckweed showed no morphological changes (Figure 2); the area of the shoots, their number, fresh and dry weight, and color showed no significant differences across the analyzed samples. However, with increased fluoride contents, there were significant decreases in chlorophyll content (Table 2) and activity of the enzymes: chlorophyllase (Chlase) and delta-aminolevulinic acid dehydratase (ALAD) (Figure 2H,I). The lowest ALAD value for soil 3 (fluoride level 4.9 mg × kg^−1^) was 98 nmol of porphobililinogen produced per 1 g of fresh weight within 1 h, while the Chlase activity for plants growing in this soil was 0.11 mmol of chlorophyllide produced per 1 g of fresh weight within 1 h. A reduction in chlorophyll content was also observed in these samples. 

Chlorophyll absorption spectra and chlorophyll concentrations calculated from the Lambert–Beer law are shown in Supplementary Figure S1. 

For soil 3, the chlorophyll content decreased by 57% relative to soil one (Table 2), but the reduction in chlorophyll content was not noticeable when visually comparing plant color (compare with Figure 2A–C). It should be noted that the amount of *chlorophyll a* was 3 to 4 times higher than *chlorophyll b* in all analyzed plants.

The F^−^ content in duckweed was also assessed before as well as after culture in the medium. It should be noted that the duckweed did take up F^−^ and its content in the surrounding medium decreased. The decrease in F^−^ content is shown in Table 3. 

### 2.2. Growth of Duckweed in Simulated Soil Extracts (50% MS Medium + F^−^)

To eliminate the influence of potential soil contaminants or deficiencies, other than the presence of fluorine, some plants were grown in 50% MS medium supplemented with F^−^ at concentrations identical to those detected in the soil samples. It should be noted that 21 metals were detected in the analyzed soil, among them heavy metals, but none of them exceeded the permissible levels (Table S1). Additionally, a treatment was used in which the fluoride concentration was increased 100-fold compared to the soil with the lowest fluoride content and a concentration of 4 mg × L^−1^ was additionally applied; 50% Murashige–Skoog medium (50% MS) was used for duckweed, as it has been used successfully by other researchers to test various types of environmental contaminants [68,69,70].

**Figure 3:**
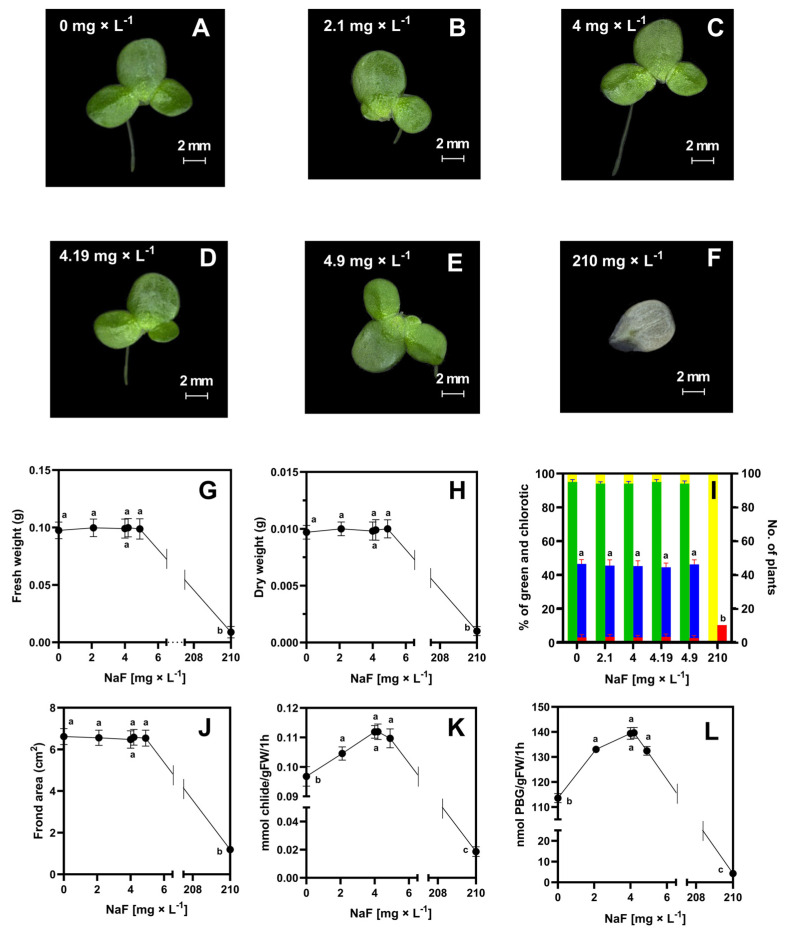
General appearance of plants after 7 days of growth in simulated soil extracts (50% MS medium + F^−^)—(**A**–**F**); fresh weight [g]—(**G**); dry weight [g]—(**H**); percent frequency of green plants (green bar), percent frequency of yellow fronds (yellow bar), number of green plants (blue bar), number of yellow plants (red bar)—(**I**); surface area [cm^2^]—(**J**); chlorophyllase activity [mmol of chlorophyllide produced per 1 g of fresh weight within 1 h]—(**K**) and aminolevulinic acid dehydratase activity [as nmol of porphobilinogen produced per 1 g of fresh weight within 1 h]—(**L**). Tests were conducted in triplicates. The one-way analysis of variance (ANOVA) was carried out using Tukey’s post hoc test (*p* ≤ 0.05). Small letters represent groups of significant difference.

Plants grown on F^−^-supplemented 50% MS media showed no morphological differences from plants grown on soil extracts with the same F^−^ concentrations; plant number, surface area, and fresh and dry weight were identical in both types of treatments. Different concentrations of fluorine also did not result in clear disturbances in plant appearance, except for the highest F**^−^** concentration, which clearly caused plant death (Figure 3). At the highest fluorine concentration (210 mg × L^−1^, not actually observed in studied soils), plants did not increase in number from the first day of culture, and their shoots disintegrated into individual segments, which were totally chlorotic. Also, the ALAD and Chlase enzymes in plants grown in 50% MS medium + fluorine showed similar activity patterns to those from plants grown in soil extracts. An almost twofold increase in fluoride concentration from 2.10 to 4.19 or 4.90 mg × L^−1^ was not toxic to the plants. The only change that was observed was a reduction in chlorophyll content, but even these changes were not detectable visually but only by spectrophotometric determinations (Table 3). 

The chlorophyll decay profile was identical in plants grown on soil extracts to the corresponding MS + fluorine plants with the same F^−^ concentration. At a fluoride ion dose of 210 mg × L^−1^, chlorophyll was present in trace amounts (0.02 × 10^−7^ M) and a reduction in chlorophyll content was visually apparent. Chlorophyll interactions with heavy metals have been studied repeatedly in terrestrial as well as aquatic plants [71,72,73,74]. An important feature of the action of metals, including heavy metals, is the substitution of Mg^2+^ ions in the porphyrin ring. Grajek et al. [71] indicated that after magnesium replacement with cadmium, a 50% decrease in chlorophyll content is observed due to the formation of the Cd-Chl complex. A decrease in fluorescence intensity and quenching by the Cd-Chl complex was also observed. Fluoride ions cannot replace Mg^2+^, and therefore, no color change was observed. 

Plants growing in 50% MS medium took up nearly 100% of F^−^ in the medium (Table 4), similar to plants grown in soil extracts. Only at the highest F^−^ concentration in 50% MS did the plants take up no more than 30% of F^−^ from the medium, and still it resulted in the death of the plants. 

### 2.3. Extracellular Reaction of Pure Chlorophyll a with Fluoride

**Figure 4:**
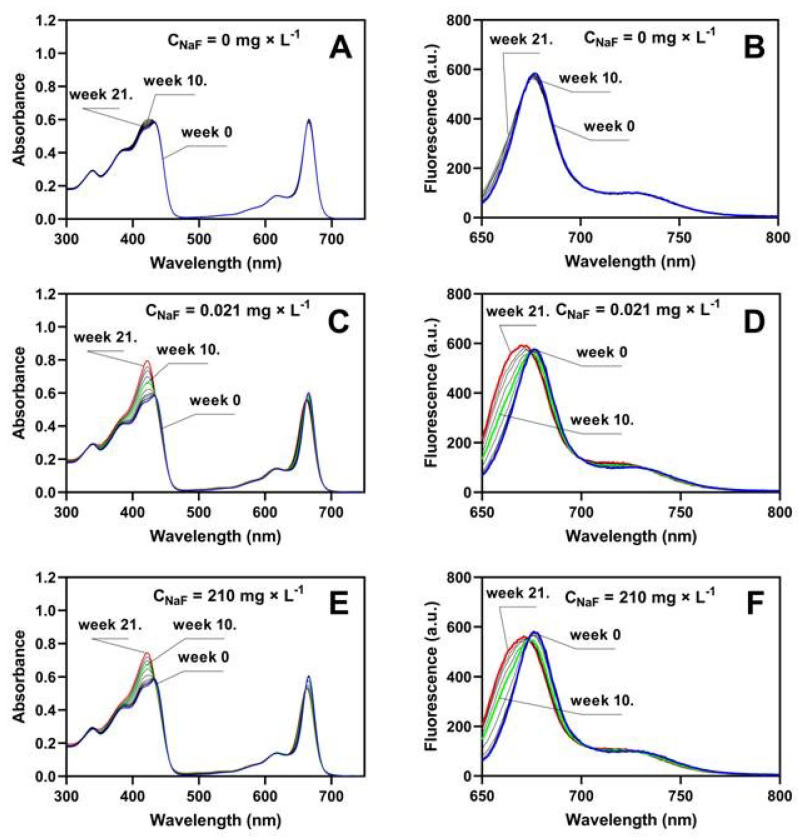
Absorption (**A**) and fluorescence (**B**) of *chlorophyll a* without NaF or with 0.021 mg × L^−1^ NaF (**C**,**D**), or with 210 mg × L^−1^ NaF (**E**,**F**). In the figures, the blue line marks the beginning of the reaction, the green line marks the 10th week, and the red line marks the 21st week of the reaction of chlorophyll with fluorine ions.

**Figure 5:**
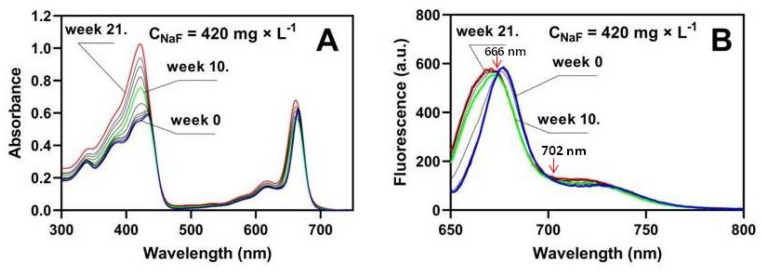
Absorption (**A**) and fluorescence (**B**) spectra of *chlorophyll a* after reaction with 420 mg × L^−1^ NaF. In the figures, the blue line marks the beginning of the reaction, the green line marks the 10th week, and the red line marks the 21st week of the reaction of chlorophyll with fluoride anions. The arrows show two isosbetic points at 666 and 702 nm.

In this section, we present the results of experiments on the effect of sodium fluoride on pure (commercial) chlorophyll using spectroscopic methods (absorption and fluorescence). Chlorophylls exhibit two main light absorption bands: the Soret band from 360 to 440 nm and the Q band consisting of Qy from 640 to 680 nm and Qx from 600 to 640 nm. The absorption and fluorescence measurement were carried out for a series of *chlorophyll a* solutions with a constant concentration of 1 × 10^−5^ M and sodium fluoride with concentrations corresponding to the fluorine content in the soil and Murashige and Skoog medium, and we additionally used a sodium fluoride concentration of 420 mg × L^−1^. The experiment was conducted for 21 weeks. We used *chlorophyll a* in our studies because its content in plants is more than three times higher than that of *chlorophyll b* [75,76,77,78,79]; our results are shown in Table 2 and Table 4. Figure 4 and Figure 5 show selected absorption and fluorescence spectra of *chlorophyll a* with the addition of NaF at concentrations of 0, 0.021, 210, and 420 mg × L^−1^. Figure 4A shows the results of *chlorophyll a* absorption tests without the addition of sodium fluoride. It can be seen that without the presence of NaF, the absorption value of *chlorophyll a* in the Q_y_ band at λ = 665 nm did not change. However, in the Soret band, the maximum chlorophyll absorption at λ = 433 nm increased slightly over the 21 weeks of the experiment. The fluorescence spectrum (Figure 4B) slightly shifted to the shortwave side after 21 weeks of storing the solutions at 4 °C and in the dark. The temperature of +4 °C was used in this study to minimize the degradation of *chlorophyll a,* and at the same time, not to significantly limit the rate of the reaction between *chlorophyll a* and sodium fluoride. It has been repeatedly shown that chlorophyll degradation is stimulated by light and temperature [56,57,80,81,82]. For short-term storage of chlorophyll (for 3–4 weeks), the recommended temperature is −20 °C, while for a longer storage, the recommended temperature is −70 °C, and it prevents a significant decrease in the concentration of *chlorophyll a* [83]. In vitro, chlorophyll molecules are not protected against photooxidation [84]. King et al. [85], however, showed that chlorophyll stored in vitro at temperatures close to 0 °C degrades much slower than at higher temperatures.

In the case of treatment of chlorophyll with different doses of NaF, the absorbance value of *chlorophyll a* decreased within 10 weeks from A = 0.602 to A = 0.577 for C_NaF_ = 0.021 mg × L^−1^ (Figure 4C), and on the 10th week there was a shift in the absorption spectrum towards the shortwave. In the Soret band, there was also a shift in the absorption spectrum towards the short wavelength, and there was a large increase in the absorption of the newly created band: from the value of A = 0.582 to A = 0.796. Two isosbestic points were visible in the absorption spectra at wavelengths λ = 662 nm and λ = 435 nm, indicating the formation of a new chlorophyll–fluoride complex. For the maximum concentration, Figure 5 shows a fully developed spectrum of the complex (red line).

By carefully analyzing the Q_y_ band for the max dose of NaF (Figure 5A), we see that until the 10th week, the absorption band for chlorophyll decreases, and then this band shifts significantly to the shortwave direction, and an increase in the newly formed band at a wavelength of λ = 672 nm is observed. The value of this band reaches its maximum at a concentration of 420 mg × L^−1^. The Soret band is changing significantly already in the 10th week. Its vibronic structure is lost and a new band of the complex is created at a wavelength of λ = 421 nm with a maximum of A = 1.026, for which the absorbance value in week 21 was almost twice as high as for *chlorophyll a* with C_NaF_ = 0.021 mg × L^−1^ (Figure 5).

**Figure 6:**
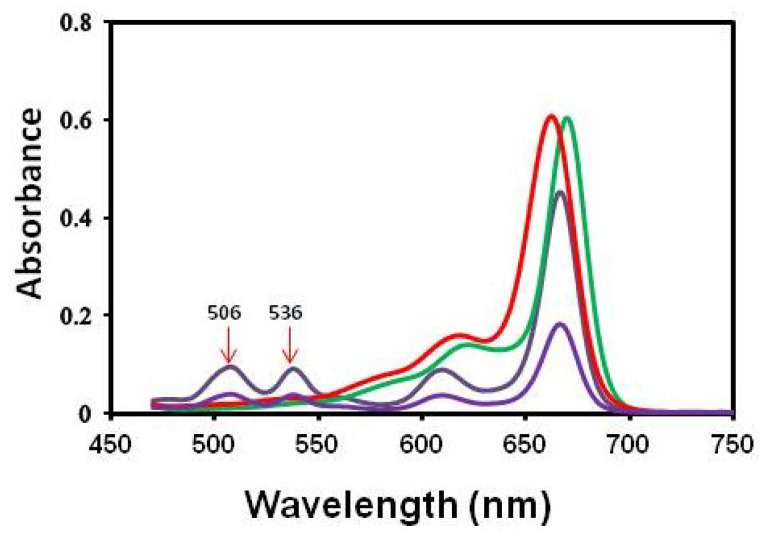
Absorption of chlorophyll (**―**), pheophytin in concentration 1 × 10^−5^ M (**―**), and a new product of chlorophyll reaction with fluoride (**―**). The arrows show two peaks characteristic for pheophytin at 506 and 536 nm.

The obtained results were fully confirmed by fluorescence tests. A strong shift of the fluorescence spectrum towards the shortwave region was observed. A new fluorescence band of the complex was created with a maximum at a wavelength of λ = 662 nm. A clear isosbestic point was visible at a wavelength of λ = 666 nm and 702 mn (Figure 5B). The reaction of HF with chlorophyll is known in the literature. However, this reaction causes the removal of magnesium and the introduction of hydrogen in its place [86,87]. In our case, we did not observe pheophytin production. The characteristic feature of the absorption spectrum of pheophytin is two absorption bands at wavelengths of 506 and 536 nm, distinguishing this compound from chlorophyll. There were no peaks at wavelengths of 506 and 536 nm, which clearly indicates the absence of pheophytin formation. Furthermore, using commercially highly purified pheophytin, we have shown that the presence of this compound does not shift the chlorophyll absorption band along the wavelength axis towards shorter wavelengths (Figure 6). We plotted pheophytin at two concentrations, *chlorophyll a*, and the new chlorophyll-fluoride complex on a single graph to compare the absorption spectra and demonstrate that there is no shift in absorption peaks concerning pheophytin.

The formation of pheophytin was observed during the reaction of tetracycline with chlorophyll, where tetracycline “extracted” magnesium from the porphyrin ring of chlorophyll, which resulted in a distinct color change chlorosis [88]. Formation of the 3F^2^ *chlorophyll a* complex was demonstrated by Ogasawara et al. [89], showing that the formation of such a complex causes a shift in the absorption spectrum by 3 nm. Our research also shows a 3 nm shift in the absorption spectra, confirming the incorporation of fluorine in the same position. We also observed a significant shift in the fluorescence spectrum of the resulting complex compared to pure chlorophyll. The fluorescence spectrum of chemical compounds shifts only when the compound reacts and forms a complex [71]. In the analyzed solution, only two chemical compounds were present: chlorophyll and fluoride, from which a new complex compound was formed. The appearance of two isosbestic points (Figure 5B) at wavelengths 666 and 702 nm indicates the equilibrium between chlorophyll and 3F^2^-chlorophyll in the solution. Based on the results obtained by Ogasawara et al. [89], we suggest that in the new complex, fluorine was attached to the 3^2^ position of chlorophyll. In our study, the formation of the fluorine derivative of chlorophyll occurred under different conditions and via a different mechanism than that observed by Ogasawara et al. [89]. In Ogasawara’s study, the reaction was conducted using pheophytin as an intermediate product; however, as mentioned, this compound was undetectable in our case, and the reaction proceeded with completely different dynamics (products visible after 21 weeks). Spectroscopic analysis, however, suggests that the final product of the reaction observed in this study was the same as that observed by Ogasawara et al. [89].

Therefore, we suggest that fluorine forms a new complex with *chlorophyll a* (Figure 4 and Figure 5); however, it does not knock out the magnesium (as is the case in plants responding to tetracycline; [88]) but it is linked to the carbon in position 3^2^ in the porphyrin ring, which results in shifts of both the absorption and fluorescence spectra. 

The paper confirms that antibiotics as environmental pollutants have a toxic effect on plant metabolism, and particularly on photosynthesis. In the case of some pharmaceuticals (e.g., tetracycline), these disorders are manifested by both restriction of growth rate and pronounced chlorosis. Our research shows that the mechanism of phytotoxic action of fluorides is different. As was found by many researchers [39,40,41,42,43,44,45,46,47,48,49,50,51], fluoride contamination results in a broad spectrum of disruptions in plants. However, the focus of this paper is on elucidating the mechanism of damage to chlorophyll molecules, as this aspect of fluorine phytotoxicity has been inadequately understood thus far. Among the analyzed F^−^ doses, only the highest concentration (210 mg × kg^−1^) resulted in pronounced chlorosis and had a lethal effect on the plants. We showed that F^−^ even at low doses results in the formation of a fluorinated derivative of chlorophyll with F^−^ linked to the carbon in the porphyrin ring in position 3^2^. We sought to investigate whether, at the highest dose of F^−^, clearly phytotoxic, its interaction with chlorophyll remains consistent with that observed at lower doses. Our findings indicate that it does.

## 3. Materials and Methods 

### 3.1. Collection of Soil and Manure Samples and Preparation of Extracts

Soil samples were taken directly from the surface layer (0–30 cm) of the arable lands in the Warmia and Masuria voivodeship in Poland in an early spring 2021. Soil 1 was from the arable field regularly fertilized with the manure and soils 2 and 3 were from the field in near the manure pile (See Figure 1). Additionally, manure samples were collected from the interior of the poultry houses and from the manure pile, located outdoors.

For each sampling site, six subsamples were collected from an area of 1 m^2^, homogenized on the site after the removal of the upper layer of organic vegetative materials, and mixed to provide a bulked sample for each site. The soils were characterized in terms of their physicochemical properties (Table 1). Particle size distribution was measured by laser diffraction method, using the Mastersizer 2000 apparatus with Hydro UM attachment (Malvern Panalytical, Malvern, UK) [90]. The pH was measured potentiometrically in a suspension in KCl solution (1 mol × L^−1^), extractable acidity was measured by Kappen method, and exchangeable cations by atomic absorption spectroscopy after soil extraction with 1 mol × L^−1^ CH_3_COONH_4_ [91].

In order to examine the effect of F pollution on common duckweed, water extracts were made from the examined soils and used as growth media. For this purpose, 100 g of each soil type was suspended in 100 mL of deionized water and shaken for 24 h on a Heidolph Unimax 1010 shaker (Heidolph, Schwabach, Germany) at 80 rpm. The samples were filtered and the content of fluoride (F^−^) in the soil extract was determined (see Section 3.3). The extracts were used as growth media for common duckweed.

### 3.2. Axenic Cultures of Duckweed (Lemna minor L.)

To eliminate other possible contaminants present in the soil, 50% Murashige and Skoog medium [92] was used as a simulated soil extract and the fluorine was added at the concentrations found in the soil extracts, at 2.1, 4.19, 4.9 mg × kg^−1^ of soil. Additionally, the content of F^−^ was increased 10 times compared to the lowest content detected in soil.

Axenic cultures of duckweed (*Lemna minor* L.), established at the Department of Plant Physiology, Genetics and Biotechnology of the University of Warmia and Mazury in Olsztyn, were used. Soil extract or 50% Murashige–Skoog (MS) medium (100 mL; supplemented with an appropriate amount of fluoride) was placed in a glass jar (a capacity of 250 mL) with 10 plants of duckweed, each with 3 visible fronds. The samples were incubated for 7 days at 16 h/8 h photoperiod and day/night temperatures of 25 °C/17 °C and with daytime light intensity of 3.4 klx (fluorescent lamp Osram L36W/77 Fluora, Osram, Munich, Germany). After 7 days of culture, plant material from each jar was tested for the activity of two enzymes: chlorophyllase (Chlase) and delta-aminolevulinic acid dehydratase (ALAD). Chlorophyll contents and morphological parameters of plants from each jar were also examined, including surface area of the fronds, fresh and dry weight, and severity of chlorosis. Chlorosis was visually assessed using a stereo microscope and by determining numbers of normal, green plants as opposed to the chlorotic plants. The experiment was carried out with three replications. 

### 3.3. Fluoride Content

Deionized water (with electroconductivity 0.05 µS × cm^−1^) from an R5 UV demineralizer (HydroLab, Straszyn, Poland) was used for preparation of fluoride standard solutions and the mobile phase. A 3.6 mM Na_2_CO_3_ solution was used as the eluent (99.95–100.05% ACS reagent, Sigma Aldrich, Buchs, Switzerland). Fluoride standards (1000 ± 2 mg × L^−1^, TraceCERT^®^ certified reference material) were obtained from Merck, Germany. Six-point linear calibration curves with three replicates were made using F^−^ standards in two ranges: 0.01–1 mg × L^−1^ and 1–40 mg × L^−1^. Samples were filtered through 0.45 µm pore size 25 mm PVDF syringe filters (ALWSCI Technologies Co., Shaoxing, China). 

An ion chromatography system with a conductometric detector (CDD-10 AVP), supplied by Shimadzu (Duisburg, Germany), was used to perform measurements. Eluent conductivity suppression was performed using a XAMS anion membrane suppressor with ASUREX-A100 automatic regenerator (Diduco AB, Umeå, Sweden). The Shodex SI-52 4E column (PEEK, 5 µm particle size, 4 mm ID × 250 mm length) from Showa Denko KK, Tokyo, Japan, was used. The eluent flow rate was 0.8 mL × min^−1^ with 30 min duration of analysis. The oven containing the column and suppressor was heated to 40 °C. The volume of each injected sample was 20 µL. The detection limit for F^−^ was 3.1 µg × L^−1^. Calibration curves for both ranges showed good linearity (R^2^ = 0.9996 and R^2^ = 0.9998, respectively). 

### 3.4. Aminolevulinic Acid Dehydratase Assay

The analysis was performed according to Jiao et al. [93]. Plant material, 0.1 g of fresh weight, was homogenized in 1 mL of 0.05 M Tris-HCl (pH 8.2) buffer containing 0.1 mM DTT. The homogenate was centrifuged at 6000× *g* for 0.5 h at 4 °C. The supernatant was used to measure enzymatic activity. Extract (1 mL) was added to 1.35 mL of 0.05 M Tris-HCl buffer (pH 8.2) containing 0.1 mM DTT, 0.08 mL of 0.2 M MgCl_2_, and 0.27 mL of 1 mg × mL aminolevulinic acid solution. The mixture was incubated for 1 h at 37 °C. Subsequently, 0.3 mL of 3 M TCA was added to stop the reaction. The sample was centrifuged at 6000× *g* for 10 min. The supernatant was mixed with Ehrlich reagent added in 1:1 (*v*/*v*) ratio, and after 10 min, the absorbance was measured at 555 nm. The molar absorption coefficient for PBG is 6.1 × 10^−4^. The activity of the enzyme was expressed as nmol of porphobilinogen produced per 1 g of fresh weight within 1 h.

### 3.5. Chlorophyllase Assay

The analysis was carried out according to Zhang et al. [94]. The plant material was homogenized in a mortar in liquid nitrogen. Plant material (1 g of fresh weight) was added to 2 mL of extraction buffer composed of 0.1 M sodium-phosphate buffer (pH 6.0) with 0.2% (*v*/*v*) Triton X-100, 30 g × L^−1^ PVPP, 1 mM PMSF and 5 mM cysteine. The homogenate was centrifuged at 9000× *g*, 4 °C for 20 min. The supernatant was used to measure enzymatic activity. Plant extract (0.2 mL) was added to 1 mL of 0.1 M sodium-phosphate buffer (pH 7.0) with 0.15% Triton X-100 and 0.2 mL of *chlorophyll a* solution in acetone. The mixture was incubated for 1 h at 40 °C, after which the reaction was completed by adding 3 mL of hexane. The sample was stirred to produce an emulsion, after which 9000× *g* at 4 °C was centrifuged for 2 min. Chlorophyllase activity was tested by measuring the absorbance of the lower aqueous phase after centrifugation at 667 nm. The molar absorption coefficient for chlorophyllide in acetone is 76.79 mM^−1^ × cm^−1^. The enzyme activity was expressed in mmol of chlorophyllide produced per 1 g of fresh weight within 1 h.

### 3.6. Chlorophyll Content Assay

Plants (fresh weight 300 mg) were homogenized in 5 mL methanol. The samples were centrifuged at 7000× *g* for 5 min. The supernatant was collected and diluted 5× in methanol. The absorbance of extract was determined using Cary 5000 UV-Vis NIR spectrophotometer. Chlorophyll concentrations were calculated based on the Lambert–Beer law, using the known molar extinction coefficient of *chlorophyll a* in methanol (ε = 66,600 M^−1^cm^−1^) [95]. Additionally, the content of *chlorophyll a* and *b* was determined using the formulas provided by Lichtenthaler and Buschmann [96] for methanol solutions of chlorophyll. However, absorbance values measured at A652 and 665 nm were substituted for A652.4 and A665.2, respectively, in the Lichtenthaler and Buschmann formulas. This discrepancy arose because our spectrophotometer was unable to set wavelength values containing fractions. The obtained values in μg × mL^−1^ were then converted to mol × L^−1^.

Chl a (μg/mL) = 16.72 A_665.2_ − 9.16 A_652.4_

Chl b (μg/mL) = 34.09 A_652.4_−15.28 A_665.2_

### 3.7. Effect of Fluoride on Chlorophyll a

*Chlorophyll a* (Sigma) was dissolved in methanol (ChemPur, Karlsruhe, Germany) to the concentration of 1 × 10^−5^ M and one of 3 concentrations of NaF, matching those in previously tested soils and MS media. Samples were incubated at room temperature in darkness for 10 days. Each day, spectrophotometric assay was performed using Cary 5000 UV-Vis NIR spectrophotometer for absorbance measurements and Cary Eclipse spectrophotometer for fluorescence measurements. Similarly, pheophytin (from ChromaDex, Longmont, CO, USA) was dissolved in methanol at concentrations of 1 × 10^−5^ M and its absorption was measured. All measurements were performed at temperatures of 25 ± 1 °C. Absorbance was tested in the wavelength range from 310 to 750 nm, emission was tested from 600 to 800 nm, and excitation was tested from 300 to 750 nm.

### 3.8. Experimental Procedure and Data Presentation

Each experiment was repeated three times and involved three internal replications. The results were presented as means followed by standard deviations.

### 3.9. Statistics

The results were analyzed using the one-way ANOVA test, and differences between groups were analyzed using Tukey’s post hoc test with probability *p* ≤ 0.05. All analyses were carried out using Statistica program 11.

## 4. Conclusions

1. Manure resulting as a by-product of poultry production contains phytotoxic levels of fluorine.
2. Extracts of such contaminated soil clearly affect the growth rate rather than morphology of *Lemna minor* as an indicator plant.
3. *Chlorophyll a* turns out to be the target of phytotoxic action of fluorine on *Lemna* plants.
4. This paper postulates the molecular mechanism of chlorophyll damage induced by fluorine, which is fluorine entering the porphyrin ring at position 3^2^, and leaving magnesium ion at its central position.
5. Aquatic plants can serve as indicators of environmental pollution with fluoride, but visual assessment of their condition is not sufficient for this purpose. It is necessary to use at least simple instrumental analyses to reveal chlorophyll damage or changes in the activity of enzymes associated with its biosynthesis and catabolism. It is necessary to use at least simple instrumental analyses to reveal chlorophyll damage or changes in the activity of enzymes associated with its biosynthesis and catabolism.

## Figures and Tables

**Table 1:** The content of fluoride ions, soil texture, extractable acidity, and exchangeable calcium cation content in the analyzed soils.

Soil	F^−^ Content mg × kg^−1^	Sand, %	Silt, %	Clay, %	USDA Soil Texture Classes	pH_KCl_	Hh cmol(+) × kg^−1^	Al^3+^ g × kg^−1^	Ca^2+^ cmol(+) × kg^−1^
1	2.1	70.38	26.75	2.87	sandy loam	5.5	2.52	4.2	3.60
2	4.19	65.81	30.55	3.65	sandy loam	4.9	4.16	5.9	3.65
3	4.9	75.50	22.26	2.24	loamy sand	4.9	3.90	4.7	2.24

**Table 2:** Chlorophyll concentration in common duckweed growing in aqueous soil extracts. F^−^ [mg × L^−1^], C [M] × 10^−5^ ± SD chlorophyll content. The one-way analysis of variance (ANOVA) was carried out using Tukey’s post hoc test (*p* ≤ 0.05). Small letters represent groups of significant difference.

	*Chlorophyll a* Lambert–Beer Method	*Chlorophyll a* Lichtenthaler, Buschmann Method	*Chlorophyll b* Lichtenthaler, Buschmann Method
Content F^−^ [mg × L^−1^]	C [M] × 10^−5^ ± SD	C [M] × 10^−5^ ± SD	C [M] × 10^−5^ ± SD
2.10	6.24 ± 0.04 a	6.25 ± 0.03 a	1.82 ± 0.03 a
4.19	5.27 ± 0.03 b	5.27 ± 0.05 b	1.61 ± 0.04 b
4.90	2.68 ± 0.02 c	2.68 ± 0.01 c	0.89 ± 0.03 c

**Table 3:** Fluoride ion content before and after seven-day growth of the common duckweed. The one-way analysis of variance (ANOVA) was carried out using Tukey’s post hoc test (*p* ≤ 0.05). Small letters represent groups of significant difference.

Initial F^−^ Content [mg × L^−1^]	Final F^−^ Content [mg × L^−1^]	Reduction in F^−^ [%]
2.10	0.25 ± 0.01 a	88.0 a
4.19	0.19 ± 0.01 b	95.5 b
4.90	0.17 ± 0.004 b	96.5 b

**Table 4:** Molar concentration of chlorophyll C [M] extracted from common duckweed growing in 50% Murashige–Skoog medium. Small letters represent groups of significant difference.

	*Chlorophyll a* Lambert–Beer Method	*Chlorophyll a* Lichtenthaler, Buschmann Method	*Chlorophyll b* Lichtenthaler, Buschmann Method
Initial F^−^ Content [mg× L^−1^]	C [M] × 10^−5^ ± SD	C [M] × 10^−5^ ± SD	C [M] × 10^−5^ ± SD
0	12.25 ± 0.08 a	12.26 ± 0.08 a	3.19 ± 0.06 a
2.10	11.46 ± 0.08 b	11.48 ± 0.08 b	3.18 ± 0.06 b
4.00	11.23 ± 0.09 b	11.23 ± 0.10 b	3.04 ± 0.07 b
4.19	10.66 ± 0.02 c	10.66 ± 0.08 c	3.00 ± 0.08 b
4.90	6.51 ± 0.01 d	6.51 ± 0.08 d	1.80 ± 0.08 b
210	0 ± 0 e	0 ± 0 e	0 ± 0 e

## Data Availability

The original contributions presented in the study are included in the article.

## Supplementary Materials

The following supporting information can be downloaded at: https://www.mdpi.com/article/10.3390/molecules29102336/s1, Table S1: The content of fluoride ions and physicochemical properties of soil—A, macroelements—B and other metals—C, D; Figure S1: Absorption spectra of chlorophyll isolated from duckweed A—growing on soil extracts and—B in conditions simulating soil extracts (50% MS + F^−^ medium).

## References

[1] Singh G., Kumari B., Sinam G., Kumar N., Mallick S. (2018). Fluoride distribution and contamination in the water, soil and plants continuum and its remedial technologies, an Indian perspective—A review. Environ. Pollut..

[2] Vithanage M., Bhattacharya P. (2015). Fluoride in the environment: Sources, distribution and defluoridation. Environ. Chem. Lett..

[3] Choudhary S., Rani M., Devika O.S., Patra A., Singh R.K., Prasad S.K. (2019). Impact of fluoride on agriculture: A review on its sources, toxicity in plants and mitigation strategies. Int. J. Chem. Stud..

[4] Rudnick R.L., Gao S., Holland H.D., Turekian K.K. (2014). Composition of the continental crust. Treatise on Geochemistry.

[5] Handa B.K. (1975). Geochemistry and genesis of fluoride-containing ground waters in India. Groundwater.

[6] Jones B.F., Eugster H.P., Rettig S.L. (1977). Hydrochemistry of the lake Magadi basin, Kenya. Geochim. Cosmochim. Acta.

[7] Kabir H., Gupta A.K., Tripathy S. (2020). Fluoride and human health: Systematic appraisal of sources, exposures, metabolism, and toxicity. Crit. Rev. Environ. Sci. Technol..

[8] Ozsvath D.L. (2006). Fluoride concentrations in a crystalline bedrock aquifer Marathon County, Wisconsin. Environ. Geol..

[9] Sivasankar V., Darchen A., Omine K., Sakthivel R., Sivasankar V. (2016). Fluoride: A world ubiquitous compound, its chemistry, and ways of contamination. Surface Modified Carbons as Scavengers for Fluoride from Water.

[10] Pyle D.M., Mather T.A. (2009). Halogens in igneous processes and their fluxes to the atmosphere and oceans from volcanic activity: A review. Chem. Geol..

[11] Feng Y.W., Ogura N., Feng Z.W., Zhang F.Z., Shimizu H. (2003). The concentrations and sources of fluoride in atmospheric depositions in Beijing, China. Water Air Soil Pollut..

[12] Neal C. (1989). Fluorine variations in Welsh streams and soil waters. Sci. Total Environ..

[13] Walna B., Kurzyca I., Siepak J., Brimblecombe P., Hara H., Houle D., Novak M. (2007). Variations in the fluoride level in precipitation in a region of human impact. Acid Rain-Deposition to Recovery.

[14] Tjahyono N., Gao Y., Wong D., Zhang W., Taylor M.P., Lindsay S.J. (2011). Fluoride emissions management guide (FEMG) for aluminium smelters. Light Metals.

[15] Tylenda C.A. (2011). Toxicological Profile for Fluorides, Hydrogen Fluoride, and Fluorine.

[16] Ranjan R., Ranjan A. (2015). Sources of fluoride toxicity. Fluoride Toxicity in Animals.

[17] Fuge R. (2019). Fluorine in the environment, a review of its sources and geochemistry. J. Appl. Geochem..

[18] Emamjomeh M.M., Sivakumar M. (2009). Fluoride removal by a continuous flow electrocoagulation reactor. J. Environ. Manag..

[19] Ayoob S., Gupta A.K. (2006). Fluoride in drinking water: A review on the status and stress effects. Crit. Rev. Environ. Sci. Technol..

[20] Naseem S., Rafique T., Bashir E., Bhanger M.I., Laghari A., Usmani T.H. (2010). Lithological influences on occurrence of high-fluoride groundwater in Nagar Parkar area, Thar Desert, Pakistan. Chemosphere.

[21] Messaïtfa A. (2008). Fluoride contents in groundwaters and the main consumed foods (dates and tea) in Southern Algeria region. Environ. Geol..

[22] Kruse E., Ainchil J. (2003). Fluoride variations in groundwater of an area in Buenos Aires Province, Argentina. Environ. Geol..

[23] Czarnowski W., Wrześniowska K., Krechniak J. (1996). Fluoride in drinking water and human urine in northern and central Poland. Sci. Total Environ..

[24] Haidouti C. (1991). Fluoride distribution in soils in the vicinity of a point emission source in Greece. Geoderma.

[25] Indermitte E., Saava A., Karro E. (2009). Exposure to high fluoride drinking water and risk of dental fluorosis in Estonia. Int. J. Environ. Res. Public Health.

[26] Ogawa Y., Tokunaga E., Kobayashi O., Hirai K., Shibata N. (2020). Current contributions of organofluorine compounds to the agrochemical industry. iScience.

[27] Abbas N., Ijaz M., Shad S.A., Binyameen M. (2016). Assessment of resistance risk to fipronil and cross resistance to other insecticides in the *Musca domestica* L. (Diptera: Muscidae). Vet. Parasitol..

[28] Abbas N., Khan H.A.A., Shad S.A. (2014). Resistance of the house fly *Musca domestica* (Diptera: Muscidae) to lambda-cyhalothrin: Mode of inheritance, realized heritability, and cross-resistance to other insecticides. Ecotoxicology.

[29] Ong S.Q., Ahmad H., Jaal Z., Rus A.C. (2016). Comparative effectiveness of insecticides for use against the house fly (Diptera: Muscidae): Determination of resistance levels on a Malaysian poultry farm. J. Econ. Entomol..

[30] Scott J.G., Alefantis T.G., Kaufman P.E., Rutz D.A. (2000). Insecticide resistance in house flies from caged-layer poultry facilities. Pest Manag. Sci..

[31] Borah J., Saikia D. (2011). Estimation of the concentration of fluoride in the ground water of Tinsukia Town master plan area of the Tinsukia district, Assam, India. Arch. Appl. Sci. Res..

[32] Pena A., Silva L.J.G., Pereira A., Meisel L., Lino C.M. (2010). Determination of fluoroquinolone residues in poultry muscle in Portugal. Anal. Bioanal. Chem..

[33] Mirlean N., Roisenberg A. (2007). Fluoride distribution in the environment along the gradient of a phosphate-fertilizer production emission (southern Brazil). Environ. Geochem. Health.

[34] Hyung S.W., Lee C.H., Kim B. (2017). Development of certified reference materials for accurate determination of fluoroquinolone antibiotics in chicken meat. Food Chem..

[35] Omotoso A.B., Omojola A.B. (2015). Fluoroquinolone residues in raw meat from open markets in Ibadan, Southwest, Nigeria. Int. J. Health Animal Sci. Food Saf..

[36] Pereira A.M., Silva L.J., Rodrigues J., Lino C., Pena A. (2018). Risk assessment of fluoroquinolones from poultry muscle consumption: Comparing healthy adult and pre-school populations. Food Chem. Toxicol..

[37] Pugajeva I., Avsejenko J., Judjallo E., Bērziņš A., Bartkiene E., Bartkevics V. (2018). High occurrence rates of enrofloxacin and ciprofloxacin residues in retail poultry meat revealed by an ultra-sensitive mass-spectrometric method, and antimicrobial resistance to fluoroquinolones in *Campylobacter* spp.. Food Addit. Contam..

[38] Widiastuti R., Martindah E., Anastasia Y. (2022). Detection and dietary exposure assessment of fluoroquinolones residues in chicken meat from the districts of Malang and Blitar, Indonesia. Trop. Anim. Sci. J..

[39] Ram A., Verma P., Gadi B.R. (2014). Effect of fluoride and salicylic acid on seedling growth and biochemical parameters of watermelon (*Citrullus lanatus*). Fluoride.

[40] Pulyaevskaya M.A., Varakina N.N., Gamburg K.Z., Rusaleva T.M., Stepanov A.V., Voinikov V.K., Rikhvanov E.G. (2011). Sodium fluoride inhibits HSP synthesis in heat-stressed cultured cells of *Arabidopsis thaliana*. Russ. J. Plant Physiol..

[41] Baunthiyal M., Ranghar S. (2014). Physiological and biochemical responses of plants under fluoride stress: An overview. Fluoride.

[42] Eleftheriou E.P., Tsekos I. (1991). Fluoride effects on leaf cell ultrastructure of olive trees growing in the vicinity of the aluminium factory of Greece. Trees.

[43] Fornasiero R.B. (2001). Phytotoxic effects of fluorides. Plant Sci..

[44] Luo J., Ni D., Li C., Du Y., Chen Y. (2021). The relationship between fluoride accumulation in tea plant and changes in leaf cell wall structure and composition under different fluoride conditions. Environ. Pollut..

[45] Panda D. (2015). Fluoride toxicity stress: Physiological and biochemical consequences on plants. Int. J. Bio-Resour. Environ. Agric. Sci..

[46] Verma K.K., Verma P., Singh M., Verma C.L. (2022). Influence of fluoride phytotoxicity in germinating seedlings of *Pisum sativum*: Modeling of morpho-physiological traits. Vegetos.

[47] Banerjee A., Roychoudhury A. (2019). Fluorine: A biohazardous agent for plants and phyto remediation strategies for its removal from the environment. Biol. Plant..

[48] Junior A.M.D., Oliva M.A., Martinez C.A., Cambraia J. (2007). Effects of fluoride emissions on two tropical grasses: *Chloris gayana* and *Panicum maximum* cv. Colonião. Ecotoxicol. Environ. Saf..

[49] Ahmed S., Karamat M., Haider A., Jabeen F., Ahmad M.N., Ansari M., Zulfiqar A., Jalal A., Nizam A. (2020). Ameliorative effects of salicylic acid on dry biomass and growth of *Pisum sativum* L. under sodium fluoride stress. Fluoride.

[50] Doley D., Hill R.J., Riese R.H. (2004). Environmental fluoride in Australasia: Ecological effects, regulation and management. Clean Air Environ. Qual..

[51] Singh M., Verma K.K. (2013). Influence of fluoride-contaminated irrigation water on physiological responses of poplar seedlings (*Populus deltoides* clone-S7C15). Fluoride.

[52] Barbier O., Arreola-Mendoza L., Del Razo L.M. (2010). Molecular mechanisms of fluoride toxicity. Chem. Biol. Interact..

[53] Rhimi N., Mezghani I., Elloumi N., Nasri M., Ben Abdallah F. (2016). Morphological and anatomical responses of pear and almond trees to fluoride air pollution. Fluoride.

[54] Gadi B.R., Kumar R., Goswami B., Rankawat R., Rao S.R. (2021). Recent developments in understanding fluoride accumulation, toxicity, and tolerance mechanisms in plants: An overview. J. Soil Sci. Plant Nutr..

[55] Hong B.D., Joo R.N., Lee K.S., Lee D.S., Rhie J.H., Min S.W., Song S.G., Chung D.Y. (2016). Fluoride in soil and plant. Korean J. Agric. Sci..

[56] Hosikian A., Lim S., Halim R., Danquah M.K. (2010). Chlorophyll extraction from microalgae: A review on the process engineering aspects. Int. J. Chem. Eng..

[57] Manolopoulou E., Varzakas T., Petsalaki A. (2016). Chlorophyll determination in green pepper using two different extraction methods. Curr. Res. Nutr. Food Sci..

[58] Haddy A., Lee I., Shin K., Tai H. (2018). Characterization of fluoride inhibition in photosystem II lacking extrinsic PsbP and PsbQ subunits. J. Photochem. Photobiol. B Biol..

[59] Fan J., Chen K., Xu J., Khaldun A.B.M., Chen Y., Chen L., Yan X. (2022). Physiological effects induced by aluminium and fluoride stress in tall fescue (*Festuca arundinacea* Schreb). Ecotoxical. Environ. Saf..

[60] Saxena V., Ahmed S. (2001). Dissolution of fluoride in groundwater: A water-rock interaction study. Environ. Geol..

[61] Saxena V., Ahmed S. (2003). Inferring the chemical parameters for the dissolution of fluoride in groundwater. Environ. Geol..

[62] Kabata-Pendias A. (2011). Trace Elements in Soils and Plants.

[63] Guo Q., Wang Y., Ma T., Ma R. (2007). Geochemical processes controlling the elevated fluoride concentrations in groundwaters of the Taiyuan Basin, Northern China. J. Geochem. Explor..

[64] Jacks G., Bhattacharya P., Chaudhary V., Singh K.P. (2005). Controls on the genesis of some high-fluoride groundwaters in India. J. Appl. Geochem..

[65] Jacks G., Rajagopalan K., Alveteg T., Jönsson M. (1993). Genesis of high-F groundwaters, southern India. J. Appl. Geochem..

[66] Ozyigit I.I., Arda L., Yalcin B., Yalcin I.E., Ucar B., Hocaoglu-Ozyigit A. (2021). *Lemna minor*, a hyperaccumulator shows elevated levels of Cd accumulation and genomic template stability in binary application of Cd and Ni: A physiological and genetic approach. Int. J. Phytoremediat..

[67] Sobrino A.S., Miranda M.G., Alvarez C., Quiroz A. (2010). Bio-accumulation and toxicity of lead (Pb) in *Lemna gibba* L. (duckweed). J. Environ. Sci. Health-Toxic/Hazard. Subst. Environ. Eng..

[68] Kim Y., Hyun S.H., Park H.E., Choi H.K. (2012). Metabolic profiling, free-radical scavenging and tyrosinase inhibitory activities of *Lemna minor* whole plants cultivated in various concentrations of proline and sucrose. Process Biochem..

[69] Krupka M., Michalczyk D.J., Žaltauskaitė J., Sujetovienė G., Głowacka K., Grajek H., Wierzbicka M., Piotrowicz-Cieślak A.I. (2021). Physiological and biochemical parameters of common duckweed *Lemna minor* after the exposure to tetracycline and the recovery from this stress. Molecules.

[70] Stefaniak B., Woźny A., Budna I. (2002). Callus induction and plant regeneration in *Lemna minor* L.. Biol. Plant..

[71] Grajek H., Rydzyński D., Piotrowicz-Cieślak A., Herman A., Maciejczyk M., Wieczorek Z. (2020). Cadmium ion-chlorophyll interaction–Examination of spectral properties and structure of the cadmium-chlorophyll complex and their relevance to photosynthesis inhibition. Chemosphere.

[72] Küpper H., Küpper F., Spiller M. (1996). Environmental relevance of heavy metal substituted chlorophylls using the example of water plants. J. Exp. Bot..

[73] Küpper H., Parameswaran A., Leitenmaier B., Trtílek M., Setlík I. (2007). Cadmium-induced inhibition of photosynthesis and long-term acclimation to cadmium stress in the hyperaccumulator *Thlaspi caerulescens*. New Phytol..

[74] Paunov M., Koleva L., Vassilev A., Vangronsveld J., Goltsev V. (2018). Effects of different metals on photosynthesis: Cadmium and zinc affect chlorophyll fluorescence in durum wheat. Int. J. Mol. Sci..

[75] Anderson J.M., Goodchild D.J., Boardman N.K. (1973). Composition of the photosystems and chloroplast structure in extreme shade plants. Biochim. Biophys. Acta Bioenerg..

[76] Croce R., Canino G., Ros F., Bassi R. (2002). Chromophore organization in the higher-plant photosystem II antenna protein CP26. Biochemistry.

[77] Ghirardi M.L., Melis A. (1988). Chlorophyll b deficiency in soybean mutants. I. Effects on photosystem stoichiometry and chlorophyll antenna size. Biochim. Biophys. Acta Bioenerg..

[78] Goodchild D.J., Park R.B. (1971). Further evidence for stroma lamellae as a source of photosystem 1 fractions from spinach chloroplasts. Biochim. Biophys. Acta Bioenerg..

[79] Tanaka R., Koshino Y., Sawa S., Ishiguro S., Okada K., Tanaka A. (2001). Overexpression of chlorophyllide a oxygenase (CAO) enlarges the antenna size of photosystem II in *Arabidopsis thaliana*. Plant J..

[80] Holm-Hansen O., Riemann B. (1978). Chlorophyll a determination: Improvements in methodology. Oikos.

[81] Schwartz S.J., Lorenzo T.V. (1991). Chlorophyll stability during continuous aseptic processing and storage. J. Food Sci..

[82] Wasmund N., Topp I., Schories D. (2006). Optimising the storage and extraction of chlorophyll samples. Oceanologia.

[83] Aminot A., Rey F. (2002). Chlorophyll a: Determination by spectroscopic methods. ICES Tech. Mar. Environ. Sci..

[84] Petrović S., Zvezdanović J., Marković D. (2017). Chlorophyll degradation in aqueous mediums induced by light and UV-B irradiation: An UHPLC-ESI-MS study. Radiat. Phys. Chem..

[85] King V.A.E., Liu C.F., Liu Y.J. (2001). Chlorophyll stability in spinach dehydrated by freeze-drying and controlled low-temperature vacuum dehydration. Food Res. Int..

[86] Cai H., Dong Y., Li Y., Li D., Peng C., Zhang Z., Wan X. (2016). Physiological and cellular responses to fluoride stress in tea (*Camellia sinensis)* leaves. Acta Physiol. Plant..

[87] Sharma R., Kaur R. (2018). Insights into fluoride-induced oxidative stress and antioxidant defences in plants. Acta Physiol. Plant..

[88] Rydzyński D., Piotrowicz-Cieślak A.I., Grajek H., Wasilewski J. (2019). Investigation of chlorophyll degradation by tetracycline. Chemosphere.

[89] Ogasawara S., Nakano K., Tamiaki H. (2020). Synthesis of fluorinated chlorophylls-a and their bio/physico-chemical properties. Eur. J. Org. Chem..

[90] Debaene G., Niedźwiecki J., Pecio A., Żurek A. (2014). Effect of the number of calibration samples on the prediction several soil properties at the farm-scale. Geoderma.

[91] Jaremko D., Kalembasa D. (2014). A comparison of methods for the determination of cation exchange capacity of soils/Porównanie metod oznaczania pojemności wymiany kationów i sumy kationów wymiennych w glebach. Ecol. Chem. Eng..

[92] Murashige T., Skoog F. (1962). A revised medium for the rapid growth and bioassay with tobacco tissue cultures. Physiol. Plant..

[93] Jiao L., Ding H., Wang L., Zhou Q., Huang X. (2017). Bisphenol A effects on the chlorophyll contents in soybean at different growth stages. Environ. Pollut..

[94] Zhang X., Zhang Z., Li J., Wu L., Guo J., Ouyang L., Xia Y., Huang X., Pang X. (2011). Correlation of leaf senescence and gene expression/activities of chlorophyll degradation enzymes in harvested Chinese flowering cabbage (*Brassica rapa* var. *parachinensis*). J. Plant Physiol..

[95] Seely G.R., Jensen R.G. (1965). Effect of solvent on the spectrum of chlorophyll. Spectrochim. Acta.

[96] Lichtenthaler H.K., Buschmann C. (2001). Chlorophylls and carotenoids. Measurement and characterization by UV-VIS spectroscopy. Curr. Protoc. Food Anal. Chem..

