# Supplementary material for: Changes in Metabolism and Content of Chlorophyll in Common Duckweed (Lemna minor L.) Caused by Environmental Contamination with Fluorides
Source: Molecules. 2024 May 16;29(10):2336. doi: 10.3390/molecules29102336 (PMC11123691; doi:10.3390/molecules29102336)
Supplement: Supplementary file 1 [file molecules-29-02336-s001.zip › molecules-2991394-supplementary.pdf]

Changes in Metabolism and Content of Chlorophyll in Common Duckweed (*Lemna minor* L.) Caused by Environmental Contamination with Fluorides

Jan Kamiński[1], Alicja Stachelska-Wierzchowska[2], Dariusz J. Michalczyk[1], Agnieszka Klimkowicz-Pawlas[3], Ewa Olkowska[4], Lidia Wolska[4], Agnieszka I. Piotrowicz-Cieślak[1,*]

[1] Department of Plant Physiology, Genetics and Biotechnology, University of Warmia and Mazury, Oczapowskiego Str. 1A, 10-719 Olsztyn, Poland
[2] Department of Physics and Biophysics, University of Warmia and Mazury, Oczapowskiego Str. 4, 10-719 Olsztyn, Poland
[3] Department of Soil Science Erosion and Land Protection, Institute of Soil Science and Plant Cultivation—State Research Institute, Czartoryskich Str. 8, 24-100 Puławy, Poland
[4] Department of Environmental Toxicology, Faculty of Health Sciences, Medical University of Gdansk, Dębowa Str. 23A, 80-204 Gdansk, Poland

*Correspondence: acieslak@uwm.edu.pl

Supplementary material

Table S1. The content of fluoride ions and physicochemical properties of soil – A, macroelements – B and other metals – C, D.

A

**Soil**	**F– Content** $mg \times kg^{-1}$	pH_{H2O}	pH_{KCl}	Hh, $cmol(+) \times kg^{-1}$	EC, $mS \times m^{-1}$	Corg, %	TC, %	TN, %	CEC, $cmol(+) \times kg^{-1}$
1	**2.1**	6.5	5.5	2.52	6.43	1.09	1.29	0.135	6.36
2	**4.19**	6.1	4.9	4.16	5.88	1.37	1.73	0.167	8.47
3	**4.9**	6.2	4.9	3.90	7.71	1.28	1.52	0.133	7.44

B

**Soil**	$N\text{-}NO_3$ $mg \times kg^{-1}$	$N\text{-}NO_4$ $mg \times kg^{-1}$	P, %	K, $mg \times kg^{-1}$	Ca, $mg \times kg$	Mg, $mg \times kg$	Ca^{2+}, $cmol(+) \times kg^{-1}$	Mg^{2+}, $cmol(+) \times kg^{-1}$	K_2O $mg \times 100g^{-1}$	K^+, $cmol(+) \times kg\text{-}^1$	P_2O_5 $mg \times 100g^{-1}$
1	4.27	5.06	0.062	1177.87	1249.26	975.91	2.77	0.45	38.4	0.61	24.8
2	3.48	3.03	0.110	1044.87	1302.09	1036.89	2.72	0.78	45.8	0.78	25.4
3	11.49	34.97	0.165	791.72	1602.95	766.83	2.40	0.45	43.2	0.67	40.4

C

**Soil**	$Na^{+,}$ $cmol(+) \times kg^{-1}$	Mo, $mg \times kg^{-1}$	Li, $\mu g \times kg^{-1}$	Be, $\mu g \times kg^{-1}$	Co, $\mu g \times kg^{-1}$	Cd, $\mu g \times kg^{-1}$	La, $\mu g \times kg^{-1}$	Hg, $\mu g \times kg^{-1}$	V, $mg \times kg^{-1}$	Mn, $mg \times kg^{-1}$	Cr, $mg \times kg^{-1}$
1	0.02	0.29	3.78	0.23	2.14	0.10	9.01	19.00	9.33	226.85	6.63
2	0.02	0.24	4.68	0.27	1.96	0.16	9.99	23.08	11.09	247.66	7.72
3	0.03	0.34	3.26	0.23	1.71	0.10	7.71	18.45	9.15	257.47	6.59

D

**Soil**	Ni,	Cu,	Zn,	As,	Se,	Sn,	Sr,	Ba,	Pb,	Na,	Fe,

	mg×kg⁻¹	mg×kg⁻¹	mg×kg⁻¹	mg×kg⁻¹	mg×kg⁻¹	mg×kg⁻¹	mg×kg⁻¹	mg×kg⁻¹	mg×kg⁻¹	mg×kg⁻¹	mg×kg⁻¹
1	4.46	5.05	31.43	1.89	0.15	0.99	6.21	29.80	9.40	60.27	5465.99
2	4.80	5.92	40.03	2.30	0.17	1.10	6.79	39.13	12.20	54.09	5751.74
3	3.81	4.08	27.74	1.97	0.14	0.69	5.29	28.00	13.63	85.89	4931.30

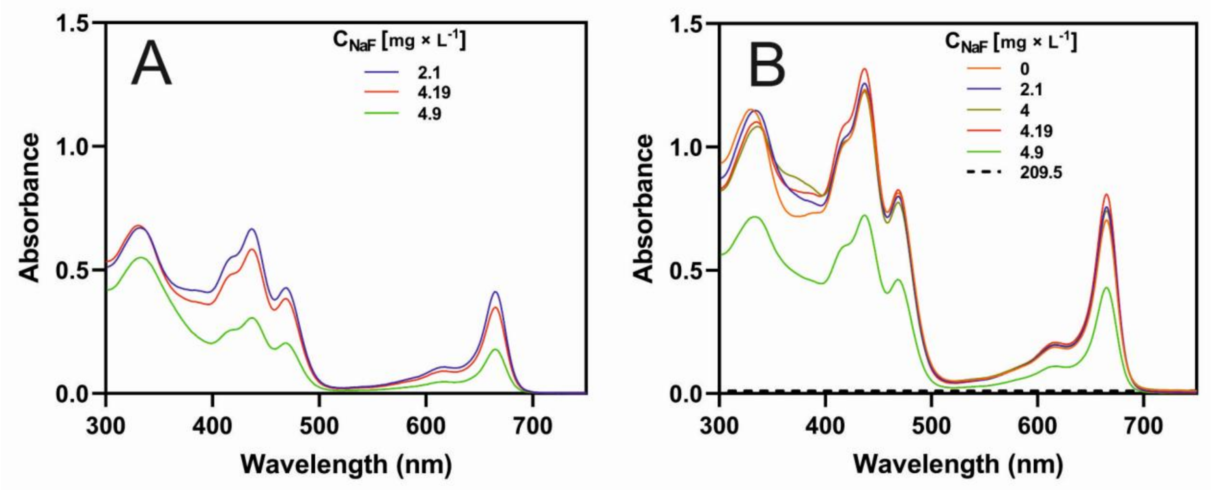

Figure S1. Absorption spectra of chlorophyll isolated from duckweed A – growing on soil extracts and – B in conditions simulating soil extracts (50% MS + F^- medium).
